# Literary Notes

**Published:** 1896-11

**Authors:** 


### Literary Notes.

The editors of *Mathews' Medical Quarterly* announce that with the January issue of that publication its name will be changed to *Mathews' Quarterly Journal of Rectal and Gastro-intestinal Diseases*. It is essential that the title of a medical journal should convey to the reader an idea of its contents, hence this change of name.

It is still the only English publication devoted to diseases and surgery of the rectum and gastro-intestinal tract, and the articles which will appear in it will be limited to these subjects. The *Journal* will continue to be edited by Drs. J. M. Mathews and Henry E. Tuley and published at Louisville, Ky.

